# Abnormal dynamic functional network connectivity in male obstructive sleep apnea with mild cognitive impairment: A data-driven functional magnetic resonance imaging study

**DOI:** 10.3389/fnagi.2022.977917

**Published:** 2022-10-25

**Authors:** Haijun Li, Lan Li, Kunyao Li, Panmei Li, Wei Xie, Yaping Zeng, Linghong Kong, Ting Long, Ling Huang, Xiang Liu, Yongqiang Shu, Li Zeng, Dechang Peng

**Affiliations:** ^1^Medical Imaging Center, The First Affiliated Hospital of Nanchang University, Nanchang, China; ^2^PET Center, The First Affiliated Hospital of Nanchang University, Nanchang, China; ^3^Department of Infection Management, Jiangxi Provincial Maternal and Child Health Hospital, Nanchang, China

**Keywords:** obstructive sleep apnea, dynamic functional connectivity, mild cognitive impairment, independent component analysis, brain network

## Abstract

**Objective:**

The purpose of this study was to investigate the dynamic functional network connectivity (FNC) and its relationship with cognitive function in obstructive sleep apnea (OSA) patients from normal cognition (OSA-NC) to mild cognitive impairment (OSA-MCI).

**Materials and methods:**

Eighty-two male OSA patients and 48 male healthy controls (HC) were included in this study. OSA patients were classified to OSA-MCI (*n* = 41) and OSA-NC (*n* = 41) based on cognitive assessments. The independent component analysis was used to determine resting-state functional networks. Then, a sliding-window approach was used to construct the dynamic FNC, and differences in temporal properties of dynamic FNC and functional connectivity strength were compared between OSA patients and the HC. Furthermore, the relationship between temporal properties and clinical assessments were analyzed in OSA patients.

**Results:**

Two different connectivity states were identified, namely, State I with stronger connectivity and lower frequency, and State II with lower connectivity and relatively higher frequency. Compared to HC, OSA patients had a longer mean dwell time and higher fractional window in stronger connectivity State I, and opposite result were found in State II, which was mainly reflected in OSA-MCI patients. The number of transitions was an increasing trend and positively correlated with cognitive assessment in OSA-MCI patients. Compared with HC, OSA patients showed extensive abnormal functional connectivity in stronger connected State I and less reduced functional connectivity in lower connected State II, which were mainly located in the salience network, default mode network, and executive control network.

**Conclusion:**

Our study found that OSA patients showed abnormal dynamic FNC properties, which was a continuous trend from HC, and OSA-NC to OSA-MCI, and OSA patients showed abnormal dynamic functional connectivity strength. The number of transformations was associated with cognitive impairment in OSA-MCI patients, which may provide new insights into the neural mechanisms in OSA patients.

## Introduction

Obstructive sleep apnea (OSA) is the most common sleep-respiratory disorder, but it is often overlooked by people (Franklin and Lindberg, 2015). The incidence of OSA in adult populations was reported to be approximately 6–38%, and was higher in the elderly and obese populations (Senaratna et al., 2017). A growing number of studies have shown that OSA is a clinical syndrome, leading to multisystem diseases, such as hypertension, osteoporosis, diabetes, depression, and anxiety, among which cognitive impairment has gradually become a concern for scholars (Vanek et al., 2020). Cognitive disorders associated with OSA mainly include disorders in memory, execution, vigilance, and so on (Olaithe et al., 2018). OSA is considered one of the potential independent risk factors for Alzheimer’s disease (Liguori et al., 2021). Therefore, the potential neural mechanisms of cognitive impairment in OSA patients should have important roles in the treatment and prevention of diseases.

Many neuroimaging studies have used magnetic resonance imaging (MRI) techniques to explore the neural basis of OSA, partly providing a theoretical basis for understanding the underlying neural mechanisms (Huang et al., 2019; Lee et al., 2019; Yan et al., 2021). In recent years, our research group has conducted a series of neuroimaging studies of OSA patients. Compared with healthy controls (HC), we found default mode network (DMN) dysfunction and frontal adaptive compensatory response in OSA patients (Li et al., 2015), and further study found that frequency-specific local spontaneous neural activity can be reversed in the temporal lobe, parietal lobe, and brainstem areas after short-term continuous positive airway pressure (CPAP) treatment (Li et al., 2021), which provided additional information on the underlying neural mechanisms of OSA-related cognitive impairment and potential neuroimaging markers for clinical treatment. In resting-state functional connectivity (FC) studies, we found abnormal FC between insular subregions and multiple other related brain regions involved in cognitive, emotional, and sensorimotor networks (Kong et al., 2022). In addition, abnormal FC between hippocampal subregions and sensorimotor network, frontoparietal network and default mode network-related brain regions were found in OSA patients (Liu et al., 2022). The local integration and integrity of brain connectivity may be disrupted from small-world networks, which can be used as quantitative physiological indicators to assist with clinical diagnosis (Chen et al., 2017). We found significantly reduced regional degree centrality values in the left middle occipital gyrus, posterior cingulate, left superior frontal gyrus and bilateral inferior parietal lobe, and increased degree centrality values in the right orbitofrontal cortex, bilateral posterior cerebellum, bilateral lenticular nucleus, hippocampus, and inferior temporal gyrus, contributing to our understanding of the neurological features of OSA at the whole-brain network level (Li H. et al., 2016). Meanwhile, after short-term CPAP treatment, OSA patients have increased degree centrality values in some brain areas such as frontal lobe, temporal lobe, and insular lobe (Li et al., 2022). CPAP treatment can effectively reverses the functional network damage caused by OSA and provides potential neuroimaging markers for CPAP treatment evaluation. Other researcher have found that OSA patients have complex and abnormal resting-state FC in different brain areas, including the cerebellum, frontal lobe, parietal lobe, temporal lobe, occipital lobe, limbic system, and basal ganglia. These abnormal FC may result in inadequate self-discipline, executive, cognitive, emotional, and sensorimotor responses (Park et al., 2016). Recently, scholars have found that patients with OSA mainly affect the cerebrocerebellar pathway, and that was associated with sleep fragmentation and hypoxia, which was thought to be involved in the cognitive decline in OSA (Park et al., 2022). The study found that after 3 months of short-term CPAP treatment, abnormal sleep, and mood scores in OSA patients significantly decreased to normal levels, meanwhile, spontaneous brain activity was decreased in autonomic and somatosensory control areas such as thalamus, putamen, posterior central gyrus, and insula, and increased in the cognitive and affective regulatory regions (Song et al., 2022). The above functional MRI (fMRI) studies were all based on the intrinsic brain activity in the resting-state of the classical frequency band, ignoring the temporal variability and failing to provide the necessary information to understand the spatiotemporal features of information processing in the human brain.

Compared with regional homogeneity, amplitude of low frequency fluctuation and FC, independent component analysis (ICA) is a data-driven processing method of resting-state fMRI, without *a priori* assumptions, it can be used to divide resting-state fMRI data into multiple functional networks and to further analyze these resting-state networks (Hu et al., 2019). The ICA methods have been widely used in neuroscience studies, such as sleep disorders (Luo et al., 2022), Parkinson’s disease (Zhou et al., 2020), Alzheimer’s disease (Soheili-Nezhad et al., 2020), etc. Zhang et al. (2013) used the ICA approach have identified seven brain networks and found that OSA specifically affects resting-state FC in cognitive and sensorimotor-related brain networks, suggesting that it is a promising tool for monitoring of neurological defects and disease progression. Some scholars have proposed that dynamic functional network connectivity (FNC) can reflect transient and periodic whole-brain temporal coupling patterns (Elton and Gao, 2015). For dynamic FNC analysis, covariance calculated across the entire sliding time window for all participants and then clustering the covariance into several brain connectivity states, can be used to explore their temporal variability and network connectivity strength (Fu et al., 2018). The dynamic FNC alterations are associated with specific cognitive states, psychiatric disorders, and neurological disorders [e.g., Alzheimer’s disease (Zhao et al., 2022), major depressive disorder (Xue et al., 2020), idiopathic generalized epilepsy (Liu et al., 2017), and Parkinson’s disease (Fiorenzato et al., 2019)], providing spatiotemporal features for understanding neural mechanisms. However, there has been no report of combining ICA with dynamic FNC to explore the neural mechanism in OSA patients.

Based on these findings, we hypothesized that the dynamic FNC and FC strength in OSA patients were changes, and altered temporal properties of the dynamic FNC in OSA was associated with the cognitive status. Therefore, we first used the ICA method to determine the resting-state functional networks. Second, we constructed dynamic FNC based on resting-state network components identified by the ICA and compared the differences between groups. Finally, the relationship between temporal properties and clinical evaluation was explored to reveal the underlying neural mechanism of cognitive impairment in patients with OSA.

## Materials and methods

### Subjects

All subjects were recruited from the sleep monitoring room at the respiratory or otolaryngology department of the First Affiliated Hospital of Nanchang University from August 2015 to June 2018. The diagnostic criteria of OSA were based on the American Academy of Sleep Medicine Clinical Practice Guideline (2007) (Collop et al., 2007). The inclusion criteria for OSA were apnea-hypopnea index (AHI) > 15/hour, male, age 20–60, and right-handedness. An AHI < 5 defined as the HC group. The exclusion criteria for OSA patients and HC were as follows: (1) other sleep disorders; (2) history of diabetes or respiratory diseases; (3) history of neurodegenerative disease, brain tumors, epilepsy, and traumatic brain injury; (4) abuse of illicit drugs or intake of psychoactive medications; and (5) MRI contraindications, such as metallic implants in the body, and claustrophobia.

### Polysomnography

Before polysomnography (PSG) monitoring, all participants were asked not to drink coffee or alcohol. All subjects underwent overnight PSG (from 22:00 to 6:00 the next morning) using the Respironics LE-Series physiological monitoring system (Alice 5 LE, Respironics, Orlando, FL, USA). PSG monitoring included a standard electrooculogram, electrocardiogram, electrocardiogram, electromyogram, snoring, body position, nasal and oral airflow, thoracic and abdominal respiratory movements, and oxygen saturation (SaO_2_), and total sleep time, sleep latency, sleep efficiency, sleep stages, arousal index, and respiratory events were recorded. See our previous study for the details (Li et al., 2021). According to the American Academy of Sleep Medicine manual, hypopnea was defined as a 30% or greater drop in airflow, lasting ≥ 10 s, accompanied by 4% or greater oxygen desaturation. Obstructive apnea was described as a continuous reduction in airflow ≥ 90% for ≥ 10 s along with evident respiratory effort. The AHI was defined as the sum of apnea and hypopnea events per hour during sleep.

### Neuropsychological assessment

All participants completed the Montreal Cognitive Assessment (MoCA) and the Epworth Sleepiness Scale (ESS) assessment. The MoCA, which assesses cognitive domains including naming, visuospatial skills, executive function, attentional, language, delayed memory, abstraction, and orientation, was used to assess cognitive function. The MoCA total score is 30, with a score below 26 was considered mild cognitive impairment, and one point is added as a correction if the years of education are less than 12 (Nasreddine et al., 2005). The ESS is a very simple questionnaire for the self-assessment of daytime sleepiness, including eight different conditions, each with a score of 0–3, and the total score ranges from 0 to 24, and a total score of more than 6 indicates drowsiness (Johns, 1991).

### Imaging data acquisition and preprocessing

All subjects were scanned using the 3.0 Tesla MRI system with an 8-channel phased-array head coil (Siemens, Munich, Germany). Before MRI scans, all subjects were told to close their eyes, not to think about anything, and not to fall asleep. First, conventional axial T2-weighted imaging and axial T1-weighted imaging were performed. Then, three-dimensional high-resolution T1-weighted images were collected. Finally, blood oxygen level-dependent fMRI data were collected using an echo-planar imaging sequence and each functional contained 240 volumes. Detailed scanning parameters are shown in Supplementary Table 1. Foam pads and earplugs were used to reduce patient head movement and scanner noise during scanning. Two senior radiologists read the images to exclude gross lesions and motion artifacts.

The Statistical Parametric Mapping (SPM12^1^) and Data Processing and Analysis for Brain Imaging (DPABI^2^) software were used to preprocess fMRI data, which were run on MATLAB 2018b (Mathworks, Natick, MA, USA). Image preprocessing mainly includes the following steps: data from DICOM to NII format; the first 10 time points was removed in order to acclimate the participants to the environment; slice timing correction and head motion correction was performed, and subjects whose head motion with maximum displacement (x, y, z) of more than 2.0 mm and maximum angular rotation (x, y, z) of more than 2.0° was excluded; three-dimensional T1 imaging was segmented into gray matter, white matter and cerebrospinal fluid with the Diffeomorphic Anatomical Registration Through Exponentiated Lie algebra (DARTEL); the rs-fMRI images were normalized to the Montreal Neurological Institute space with DARTEL and resampled to 3 mm × 3 mm × 3 mm voxels; the images were spatially smoothed using a Gaussian kernel of 6 mm full-width at half-maximum. We excluded 8 OSA patients as for head motion criterion. Finally, 82 male OSA patients and 48 male HC were included in the analysis.

### Group independent component analysis and resting-state network identification

After data preprocessing, we used the group ICA function of the fMRI Toolbox (GIFT v4.0c^3^) to decompose the data into a group-level spatial function independent component. First, principal component analysis was performed to reduce the data dimension for subject specificity. The minimum description length standard was used to automatically estimate the number of independent components (ICs) (resulting in 44 ICs) for all participants. Second, to ensure the reliability and stability of the ICs, the infomax algorithm with ICASSO was run by repeating 20 times (Wang et al., 2020). Finally, the subject-specific spatial maps and time courses were back-reconstructed using group ICA, and the results were converted to a z score for display. Based on previous studies (Allen et al., 2014; Damaraju et al., 2014) and a publicly available atlas by the Functional Imaging in Neuropsychiatric Disorders Laboratory,^4^ 25 meaningful ICs were identified and were classified into eight functional networks by visual observation of the ICA results. The detailed information and spatial maps of the ICs are listed in Supplementary Table 2 and Supplementary Figure 1.

According to Allen et al.’s (2014) study, we performed additional postprocessing on the 25 ICs to reduce the remaining noise. The 3dDespike algorithm^5^ was used for linear drift, filtered using a fifth order Butterworth filter with a 0.15 Hz high frequencies cut-off. Finally, we regressed out the movement parameters.

### Dynamic functional network connectivity analysis

The sliding window technique is the most common method to study dynamic FNC (Kim et al., 2017). We computed this analysis using the temporal dynamic FNC toolbox V1.0a in GIFT. First, a sliding time window approach with a window size set to 30 TRs with a Gaussian and steps of 1 TR was used to compute the dynamic FNC between all ICs time courses. A total of 26,000 windowed FNC matrices (130 subjects × 200 matrices) were produced. Then, the k-means clustering algorithm (using the squared Euclidean distance method with a maximum of 500 iterations and 150 replicate dynamic FNC windows) was conducted on the windowed FNC matrices (Malhi et al., 2019). To estimate the optimal number of clusters, a cluster validity analysis was performed using gap and silhouette statistics on the samples of all subjects (resulting is 2).

### Statistical analysis

For demographic and clinical data, the Kolmogorov-Smirnov test was used to test the normality of the data. One-way analysis of variance (ANOVA) was used to assess differences between the three groups, a *p* < 0.05 was considered to be statistically significant, and the *post-hoc t*-test was used to compare differences between any two groups, Bonferroni correction.

For the dynamic FNC, we investigated the temporal properties of dynamic FNC states by computing the mean dwell time and fractional windows in each state, as well as the number of transitions from one state to another. One-way ANOVA was used to compare each of the 300 mean dynamic FNC correlations (25 × 24/2) from each of the 2 states between the three groups (HC, OSA-NC, OSA-MCI), age, years of education, and head motion as covariates. In addition, three dynamic FNC indices were extracted from all two states of each subject (Jiang et al., 2020), namely, the fractional window of each state, mean dwell time, and number of transitions. Fractional window indicates the percentage of time spent in each state out of the total time, mean dwell time reflects the average length of time the subjects spent in a certain state, and number of transitions refers to the number of times a subject switched between different states. These indices were compared by the two sample *t*-test, and *p* < 0.05 was considered statistically significant. The two sample *t*-test were used to compare the connectivity strength of each state between OSA and HC (*p* < 0.01, FDR correction).

The dynamic FNC values, such as fractional window, mean dwell time, and number of transitions, were analyzed for correlation with clinical data using Pearson correlations (such as MoCA, ESS, PSG) in OSA patients. The *p* < 0.05 was considered statistically significant.

## Results

### Population and clinical characteristics

According to the grading criteria, the 82 OSA patients were divided into 41 OSA-NC and 41 OSA-MCI. All of the clinical data conformed to the normal distribution. The clinical data are shown in Table 1. One-way ANOVA showed that no significant differences were found in age, years of education, and FD, and significant differences in BMI, AHI, nadir SaO_2_, mean SaO_2_, AI, ESS, and MoCA were found between three groups. The detailed results of the *post-hoc t*-test are shown in Table 1.

**TABLE 1:** Population and clinical data in OSA patients and HC.

Characteristic	HC (*n* = 48)	OSA-NC (*n* = 41)	OSA-MCI (*n* = 41)	*F* value	*P*-value	HC vs. OSA-NC *P*-value	HC vs. OSA-MCI *P*-value	OSA-NC vs. OSA-MCI *P*-value
Age, years	41.2 ± 10.1	38.9 ± 9.8	40.4 ± 10.8	1.558	0.234	0.568	0.785	0.686
BMI, Kg/m2	20.6 ± 1.6	26.7 ± 3.8	27.1 ± 3.2	60.002	< 0.001	< 0.001	< 0.001	0.915
Education, years	11.6 ± 3.1	12.6 ± 3.3	11.2 ± 3.6	1.762	0.761	0.561	0.885	0.212
AHI, /h	2.4 ± 1.3	50.3 ± 19.0	53.2 ± 23.1	130.605	< 0.001	< 0.001	< 0.001	0.915
Nadir SaO_2_, %	92.9 ± 3.3	68.2 ± 12.7	72.2 ± 11.3	83.395	< 0.001	< 0.001	< 0.001	0.199
Mean SaO_2_, %	96.2 ± 2.3	91.3 ± 5.3	92.3 ± 3.5	20.669	< 0.001	< 0.001	< 0.001	0.799
AI, /hour	11.6 ± 2.9	34.6 ± 23.0	32.6 ± 23.8	21.194	< 0.001	< 0.001	< 0.001	0.945
ESS, scores	3.1 ± 1.5	9.6 ± 4.8	11.7 ± 4.2	67.453	< 0.001	< 0.001	< 0.001	0.033
MoCA, scores	27.3 ± 1.7	27.4 ± 1.2	22.5 ± 2.8	84.889	< 0.001	0.986	< 0.001	< 0.001
Mean FD, mm	0.20 ± 0.11	0.24 ± 0.12	0.25 ± 0.12	1.751	0.178	0.550	0.234	0.967

OSA, obstructive sleep apnea; HC, health controls; BMI, body mass index; AHI, apnea hypopnea index; SaO_2_, oxygen saturation; AI, arousal index; ESS, Epworth Sleepiness Scale; MoCA, Montreal Cognitive Assessment; FD, framewise displacement.

### Resting-state intrinsic functional network connectivity

**FIGURE 1:**
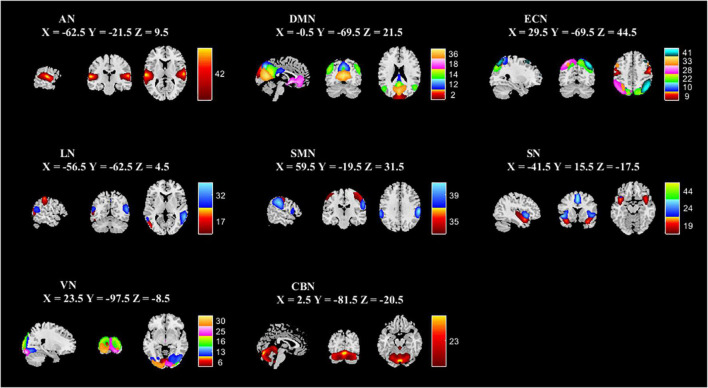
Spatial independent component analysis was used to identify independent components. Independent component spatial maps divided on eight functional networks (AN, DMN, ECN, LN, SMN, SN, VN, and CBN) based on their anatomical and functional properties. ICA, independent component analysis; AN, auditory network; DMN, default mode network; ECN, executive control network; LN, language network; SMN, sensorimotor network; SN, salience network; VN, visual network; CBN, cerebellar network.

**FIGURE 2:**
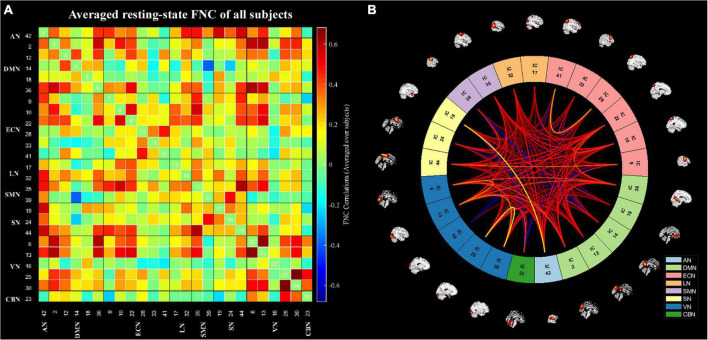
The static functional network connectivity results. **(A)** The averages static functional network connectivity matrices of all subjects between independent components pairs was produced in entire resting-state time courses. **(B)** The mean static functional network connectivity of all subjects in eight network (single sample *t*-test) (*p* < 0.01, FDR correction). AN, auditory network; DMN, default mode network; ECN, executive control network; LN, language network; SMN, sensorimotor network; SN, salience network; VN, visual network; CBN, cerebellar network.

The spatial maps of all 25 ICs are summarized in Figure 1. The functional networks were divided into the auditory network (AN) (IC42), DMN (IC2, IC12, IC14, IC18, IC36), executive control network (ECN) (IC9, IC10, IC22, IC28, IC33, IC41), language network (LN) (IC17, IC32), sensorimotor network (SMN) (IC35, IC39), salience network (SN) (IC19, IC24, IC44), visual network (VN) (IC6, IC13, IC16, IC25, IC30), and cerebellar networks (CBN) (IC23). The averaged static FNC matrix between 25 ICs of all subjects is shown in Figure 2A. The single sample *t*-test results of all subjects are shown in Figure 2B. The stronger connections were mainly located in AN, DMN, ECN, LN, SMN, SN, and VN.

### Dynamic functional network connectivity alterations

**FIGURE 3:**
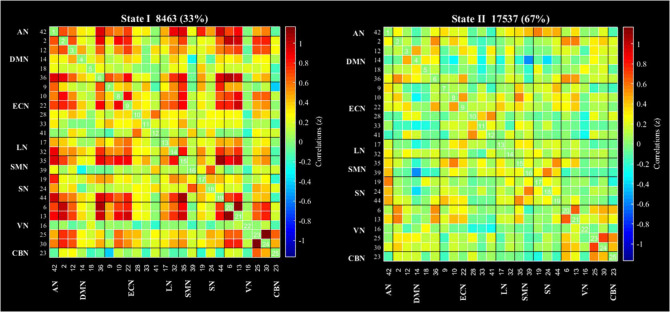
Results of the clustering analysis per state. Cluster centroids for each state of all participant. The total number of occurrences and percentage of total occurrences are listed in each cluster. AN, auditory network; DMN, default mode network; ECN, executive control network; LN, language network; SMN, sensorimotor network; SN, salience network; VN, visual network; CBN, cerebellar network.

According to the estimate results of cluster status using gap and silhouette statistic criterion, all the participants time-varying dynamic FNC was clustered into two different states by k-means clustering (*k* = 2). State I was characterized by less frequency (33%) and the presence of stronger connectivity in all networks, except for the CBN; State II was characterized by more frequency (67%) and relatively weaker connectivity (Figure 3).

**FIGURE 4:**
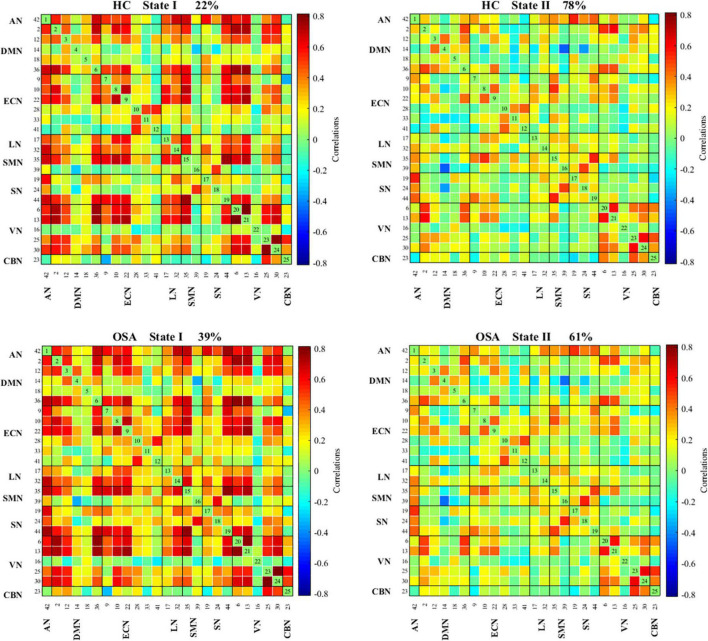
Functional connectivity state results. Group functional network connectivity matrices for each state (percentage of total occurrences for State I and II: 22 and 78% in the healthy controls and 39 and 61% in the OSA groups, respectively). There are significant statistical differences between two groups (*p* < 0.05). AN, auditory network; DMN, default mode network; ECN, executive control network; LN, language network; SMN, sensorimotor network; SN, salience network; VN, visual network; CBN, cerebellar network.

The state- and group-specific cluster center obtained by the k-means cluster analysis (*k* = 2) are shown in Figure 4. We observed that in the HC and all OSA patients, State I, integrated state with stronger connectivity within and between networks located mostly in the network (AN, DMN, ECN, LN, SMN, SN, VN); while State II, segregated state with relatively weaker connectivity within networks and anti-correlation connections between the ECN, SMN, and DMN.

**FIGURE 5:**
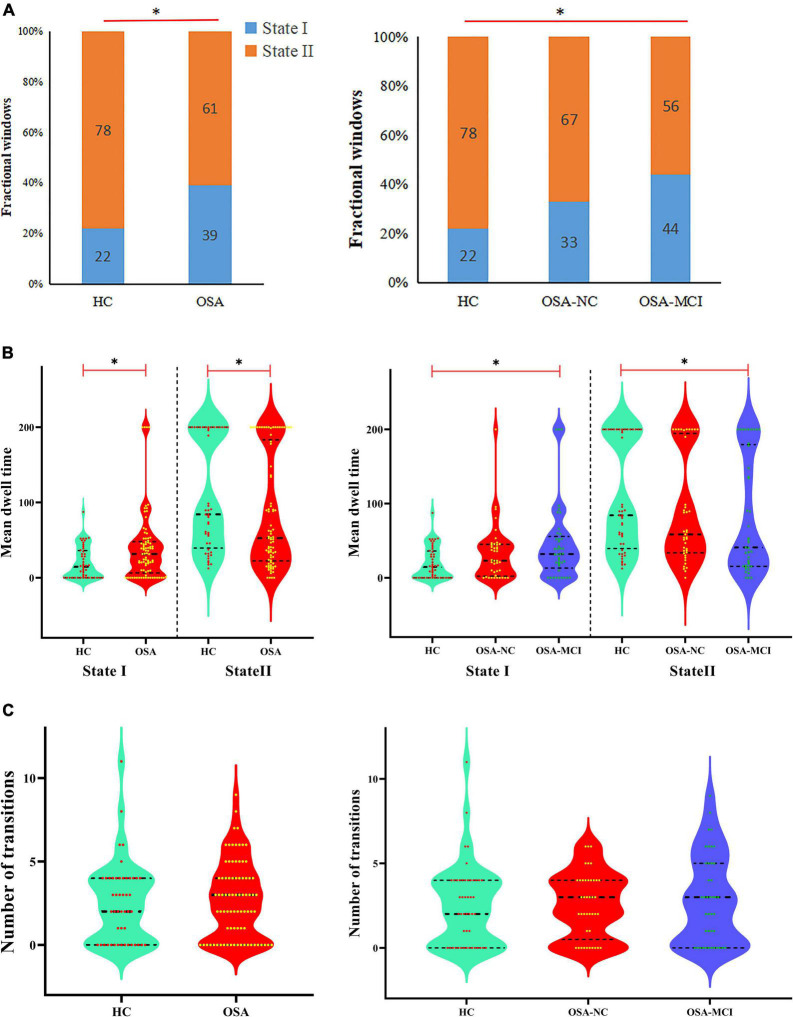
Temporal properties of dynamic functional network connectivity states for the OSA and HC groups. **(A)** Percentage of total time subjects spent in each state. **(B)** Mean dwell time and **(C)** number of transitions between states were plotted using violin plots. Horizontal black dotted lines indicate group medians and inter-quartile range. *p < 0.05. OSA, obstructive sleep apnea; HC, healthy control; MCI, mild cognitive impairment; NC, normal cognitive.

The differences of temporal properties of dynamic FNC between OSA patients and HC are shown in Table 2 and Figure 5. In OSA patients, State I occurred 17% more often than in HC, paralleled by a proportional reduction of State II. In OSA-MCI patients, State I was more frequently observed than that in HC (OSA-MCI: 0.44 ± 0.35, HC: 0.22 ± 0.26, *p* = 0.003). OSA-MCI showed a significantly longer mean dwell time in State I (OSA-MCI: 45.4 ± 51.6, HC: 19.9 ± 21.2, *p* = 0.008). However, the opposite results were shown in State II. There were no significant differences between HC and OSA-NC. The number of transitions in the three groups were 2.5 ± 2.4, 2.6 ± 1.9, 2.8 ± 2.6, respectively (*p* > 0.05).

**TABLE 2:** The dynamic FNC temporal properties between OSA patients and HC groups.

Properties	State	HC	OSA-NC	OSA-MCI	HC vs. OSA-NC *P*-value	HC vs. OSA-MCI *P*-value	OSA-NC vs. OSA-MCI *P*-value
Mean dwell time	I	19.9 ± 21.2	32.3 ± 37.1	45.4 ± 51.6	0.112	**0.008**	0.337
	II	108.7 ± 74.0	88.2 ± 72.0	82.1 ± 78.4	0.182	**0.022**	0.271
Fractional windows	I	0.22 ± 0.26	0.33 ± 0.32	0.44 ± 0.35	0.113	**0.003**	0.189
	II	0.78 ± 0.26	0.67 ± 0.32	0.56 ± 0.35	0.113	**0.003**	0.189
Number of transitions		2.5 ± 2.4	2.6 ± 1.9	2.8 ± 2.6	0.599	0.571	0.869

OSA, obstructive sleep apnea; HC, health controls; OSA-NC, obstructive sleep apnea with normal cognition; OSA-MCI, obstructive sleep apnea with mild cognitive impairment; FNC, functional network connectivity. The bold values indicates statistical significance.

**FIGURE 6:**
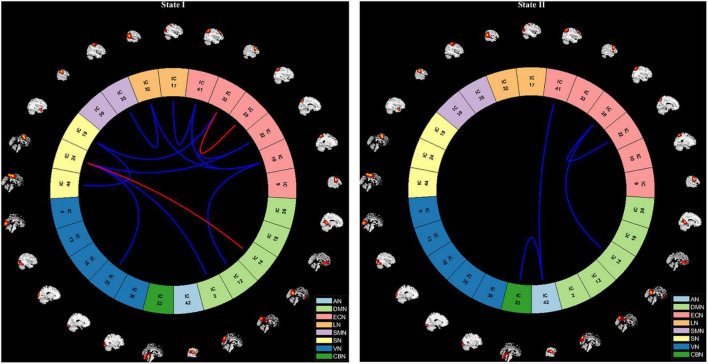
Visualization of functional network connectivity differences in two states (*p* < 0.01, FDR correction). The red line represents increased functional connectivity and the blue line represents decreased functional connectivity in OSA patients. AN, auditory network; DMN, default mode network; ECN, executive control network; LN, language network; SMN, sensorimotor network; SN, salience network; VN, visual network; CBN, cerebellar network.

We further compared the strength of connection in the two states between all OSA patients and HC groups, and the results are shown in Figure 6. In the State I, OSA patients observed 2 stronger connections and 11 lower connections compared to HC, which included 4 within-network connections (1 enhanced connections within ECN; and 3 lower connections within SN and ECN), and 9 between-networks connections (1 enhanced connections between DMN-SN, and 8 lower connections between DMN-ECN, DMN-SN, VN-SN, SN-ECN, SMN-LN, LN-ECN). In the State II, compared with HC, OSA patients showed 4 lower connections, including 1 within-network connections (ECN) and 3 between-network connections (AN-ECN, AN-CBN, and DMN-ECN).

### Correlation results

Unfortunately, no significant correlation between the dynamic FNC of temporal properties (mean dwell time, fractional windows, and number of transitions) and clinical data using Pearson correlation analysis was found in all OSA patients. However, in OSA-MCI patients, we found that the mean dwell time in State I and State II was positively correlated with the AHI and AI, the fractional windows were positively associated with the AHI and AI. The number of transitions was negatively correlated with the MoCA in OSA-MCI patients. Detailed related results are shown in Table 3.

**TABLE 3:** Correlations between temporal properties of dynamic FNC and clinical characteristics in OSA-MCI patients.

		AHI	Nadir SaO_2_	Mean SaO_2_	AI	Sleep efficiency	ESS	MoCA
Mean dwell time in State I	*r* value	0.323	–0.200	–0.257	0.383	0.006	0.020	0.058
	*p*-value	**0.040**	0.210	0.105	**0.014**	0.969	0.902	0.721
Mean dwell time in State II	*r* value	–0.325	0.210	0.160	–0.32	0.162	–0.183	0.162
	*p*-value	**0.038**	0.187	0.317	**0.041**	0.311	0.252	0.312
Fractional windows in State I	*r* value	0.363	–0.238	–0.248	0.376	–0.066	0.143	–0.143
	*p*-value	**0.020**	0.134	0.118	**0.016**	0.682	0.373	0.372
Fractional windows in State II	*r* value	–0.363	0.238	0.248	–0.376	0.066	–0.143	0.143
	*p*-value	**0.020**	0.134	0.118	**0.016**	0.682	0.373	0.372
Number of transitions	*r* value	0.139	0.013	0.156	–0.086	–0.199	0.135	–0.326
	*p*-value	0.387	0.937	0.331	0.595	0.212	0.399	**0.038**

AHI, apnea hypopnea index; SaO_2_, oxygen saturation; AI, arousal index; ESS, Epworth Sleepiness Scale; MoCA, Montreal Cognitive Assessment. The bold values indicates statistical significance.

## Discussion

In this study, we used a data-driven ICA approach to clarify the intrinsic network components in OSA patients and for the first time to explored dynamic FNC patterns in patients with OSA ranging from normal cognition to mild cognitive impairment using sliding windows and the k-means clustering method. Our main findings were as follows: (1) Two different connectivity states were determined in all subjects, namely, State II, segregated state with weaker connectivity and high frequency, State I, integrated state with stronger connectivity and low frequency; (2) Compared with HC, OSA-MCI patients appear more frequent and had longer mean dwell time in stronger connected State I, and lower connected State II appears less frequently and has a shorter mean dwell time; However, there was no significant difference between the OSA-NC and HC. In OSA-MCI patients, there was a trend to increase in number of transitions, and was positively correlated with cognitive assessment; (3) OSA patients showed more abnormal FC in stronger connected State I and showed less reduced FC in lower connected State II. These results suggested that alterations in dynamic FC patterns were associated with the presence of MCI in OSA, providing new insights into understanding neurocognitive mechanisms in patients with OSA.

In this study, we found that the incidence of lower connectivity in State II in OSA patients was lower than that in HC, along with reduced mean dwell time, while the incidence of stronger connectivity in State I was higher than that in HC. In previous studies of acute sleep deprivation, increased subcortical-cortical frequency was found after acute sleep deprivation, instead, another low connectivity state significantly reduced frequency, reflecting changes in the dynamics of mental activity in the brain after sleep loss (Li et al., 2020). Other studies found that patients with Parkinson’s disease-MCI had significantly reduced time spent in state characterized by low connectivity compared with HC, while these differences were not found in Parkinson’s disease-NC patients, suggesting that dynamic FC patterns may be related to the presence of MCI in Parkinson’s disease patients (Díez-Cirarda et al., 2018). Previous studies of dynamic FC in patients with schizophrenia found that medication-naive schizophrenia patients had shorter mean dwell time and fewer fractional windows in sparse connectivity, and longer mean dwell time and more fractional windows in intermediate connectivity, suggesting associations with brain network temporal dynamics (Lottman et al., 2017). This study analyzed the changes in dynamic FNC patterns in OSA patients from normal cognition to mild cognitive impairment. The results showed that the frequency of State II characterized by lower connectivity was 22% lower in OSA-MCI patients than that in HC, but not in the OSA-NC group, indicating that OSA patients need higher network integration. Meanwhile, we found that the mean dwell time and fractional windows in State I and II were positively associated with the AHI and AI in OSA-MCI patients, which suggested that the temporal variability of the brain functional network was related to disease severity in OSA patients.

Furthermore, compared to the HC group, there was no significant difference in the number of transitions between the states in OSA patients, but there was a tendency toward an increase in the number of transitions in OSA from normal cognition to mild cognitive impairment. Previous studies on Parkinson’s disease found no significant difference in status transition in Parkinson’s disease patients without diagnosed MCI compared to HC (Kim et al., 2017), but showed a significantly increased number of transitions in patients with Parkinson’s disease-MCI (Díez-Cirarda et al., 2018), which indicated that gradual dysfunctional patterns may exist in Parkinson’s disease patients. Another study of major depressive disorder found no significant difference but a slight increase in the number of transitions in major depressive disorder patients compared with HC (Yao et al., 2019). The results of this study were similar to findings on Parkinson’s disease and major depressive disorder. In OSA-MCI patients, increasing the number of transitions was positively correlated with MoCA score, indicating that dynamic network conversion may provide potential neural markers for cognitive deterioration in OSA.

We further compared the strength of connections in different states between OSA patients and the HC. We observed that compared with HC, OSA patients showed extensive abnormal FC in stronger connected State I and less reduced FC in lower connected State II, mainly in intranetwork connectivity (SN and ECN) and internetwork connectivity (mainly between DMN, ECN, and SN). Human cognitive function relies on efficient coordination between multiple interacting large-scale functional brain networks, in which the ECN, DMN, and SN are core networks for high-level cognitive activity. The SN is mainly composed of the dorsal anterior cingulate cortex and insular cortex, and is used to identify the most relevant stimuli in internal and external stimuli and switch to the corresponding network processing, while also participating in various cognitive functions, such as attention control, conflict monitoring, error monitoring and detection (Kerns et al., 2004; Seeley et al., 2007). The ECN is mainly composed of the dorsolateral prefrontal cortex and the posterior parietal cortex, which plays an important role in emotional and adaptive cognitive control. The DMN includes medial prefrontal cortex and posterior cingulate cortex, which are mainly responsible for autobiographical memory and self-reference processing, and episodic memory. Studies have shown that an important function of the SN is to adjust the flexible interactive switching between the activation and withdrawal of the DMN and the ECN (Sridharan et al., 2008). Specifically, in a self-related psychological task, SN induces DMN activation and ECN inactivation, while in a cognitive demands task, SN induced activation of ECN and inactivation of DMN (Chiong et al., 2013). Previous literature has described alterations in ECN activation and DMN inactivation during working memory tasks in patients with OSA (Prilipko et al., 2011). Previous studies found significant positive FC between the SN and ECN, and negative FC between the SN and DMN. This selective impairment of resting-state FC between the SN and the DMN, which may be the potential basis for cognitive impairment in OSA patients (Zhang et al., 2015). Studies have also shown the functional separation of the ECN and DMN brain regions in OSA patients (Harper et al., 1985). Our previous study found reduced DMN network connectivity and topological reorganization in patients with OSA (Li H. J. et al., 2016; Chen et al., 2018), which may underlie the cognitive deficits in patients with OSA. Some scholars have also found that the FC of the SN, bilateral ECN and DMN in OSA patients is decreased, which is related to autonomic disorders, suggesting that autonomic dysfunction in OSA is associated with central autonomic network alterations (Lin et al., 2020). Similar to these findings, this study found that OSA patients had lower connections within the SN and ECN, and internetwork connections between the DMN, ECN and SN, indicating that FNC was separated and integration ability decreased, which may be the basis of advanced cognitive impairment in OSA patients, and further supplemented the mechanism of cognitive impairment in OSA from a dynamic perspective of OSA patients.

Moreover, our study also found that VN-SN, SMN-LN, LN-ECN, AN-ECN, and AN-CBN network connections were decreased in OSA patients. The AN, SMN, VN, and LN are low-level perceptual networks that mainly receive external stimuli and play a central role in information transmission in the external environment (Shang et al., 2014). The reduced FC between the VN and the SN may reflect that the OSA exhibits poor visual information processing power. The reduced functional network of SMN-LN, LN-ECN, and AN-ECN indicate decreased receptivity to external stimuli, which affects higher cognitive function.

Some limitations should be considered in our study. First, due to gender differences in outpatients, we collected more male OSA patients, while female and children OSA patients were collected in smaller numbers, so female and children patients were excluded from our analysis. It is difficult to generalize this conclusion to the whole OSA population. Second, similar to most ICA studies thus far that have focused on gray matter, while ignoring the physiological significance of white matter signals, our study only explored changes in the temporal properties of dynamic FNC in gray matter. However, recent studies have confirmed that white matter fMRI signals contain rich spatiotemporal information similar to those found in gray matter (Wang et al., 2022). Finally, our study is only a cross-sectional study, and it is not clear how CPAP therapy affects the dynamic FNC attributes in OSA patients. In the future, female and children with OSA should be included, the dynamic FNC of white matter brain regions may provide additional information from whole brain network, and longitudinal studies would provide more information for clinical treatment.

## Conclusion

This study was the first to use the combined ICA and dynamic FNC methods to investigate dynamic temporal properties in OSA patients from normal cognition to mild cognitive impairment. In OSA-MCI patients, we found that temporal properties of dynamic FNC (mean dwell time and fractional window) were altered, and number of transformations were associated with cognitive state. It was also found that OSA patients showed abnormal high-level cognitive network connections in the stronger connected State I, mainly between the SN, DMN, and ECN. These abnormalities may be neuroimaging mechanisms in OSA-MCI patients, providing new insights into our understanding of cognitive impairment in OSA patients, and future studies should consider using dynamic FNC to predict MCI in OSA.

## Data availability statement

The raw data supporting the conclusions of this article will be made available by the authors, without undue reservation.

## Ethics statement

This study was approved by the Medical Ethics Committee of the First Affiliated Hospital of Nanchang University. The participants signed written informed consent forms, in accordance with the Declaration of Helsinki. The patients/participants provided their written informed consent to participate in this study.

## Author contributions

DP guided and designed the MRI experiment. HL analyzed the resting-state fMRI data. LL performed statistical analysis. HL and LL wrote the manuscript. KL, PL, WX, YZ, LK, TL, LH, XL, LZ, and YS collected the resting fMRI data and clinical data. HL and DP reviewed and revised the manuscript. All authors contributed to the article and approved the submitted version.

## Footnotes

1
https://www.fil.ion.ucl.ac.uk/spm/software/spm12/

2
http://rfmri.org/dpabi

3
https://trendscenter.org/software/gift/

4
http://findlab.stanford.edu/functional_ROIs.html

5
http://afni.nimh.nih.gov/afni

## References

[B1] Allen E. A. Damaraju E. Plis S. M. Erhardt E. B. Eichele T. Calhoun V. D. (2014). Tracking whole-brain connectivity dynamics in the resting state. *Cereb. Cortex* 24 663–676. 10.1093/cercor/bhs352 23146964 PMC3920766

[B2] Chen L. T. Fan X. L. Li H. J. Nie S. Gong H. H. Zhang W. (2017). Disrupted small-world brain functional network topology in male patients with severe obstructive sleep apnea revealed by resting-state fMRI. *Neuropsychiatr. Dis. Treat.* 13 1471–1482. 10.2147/NDT.S135426 28652747 PMC5473494

[B3] Chen L. Fan X. Li H. Ye C. Yu H. Gong H. (2018). Topological reorganization of the default mode network in severe male obstructive sleep apnea. *Front. Neurol.* 9:363. 10.3389/fneur.2018.00363 29951028 PMC6008385

[B4] Chiong W. Wilson S. M. D’Esposito M. Kayser A. S. Grossman S. N. Poorzand P. (2013). The salience network causally influences default mode network activity during moral reasoning. *Brain* 136 1929–1941. 10.1093/brain/awt066 23576128 PMC3673466

[B5] Collop N. A. Anderson W. M. Boehlecke B. Claman D. Goldberg R. Gottlieb D. J. (2007). Clinical guidelines for the use of unattended portable monitors in the diagnosis of obstructive sleep apnea in adult patients. Portable monitoring task force of the American academy of sleep medicine. *J. Clin. Sleep Med.* 3 737–747. 18198809 PMC2556918

[B6] Damaraju E. Allen E. A. Belger A. Ford J. M. McEwen S. Mathalon D. H. (2014). Dynamic functional connectivity analysis reveals transient states of dysconnectivity in schizophrenia. *Neuroimage Clin.* 5 298–308. 10.1016/j.nicl.2014.07.003 25161896 PMC4141977

[B7] Díez-Cirarda M. Strafella A. P. Kim J. Peña J. Ojeda N. Cabrera-Zubizarreta A. (2018). Dynamic functional connectivity in Parkinson’s disease patients with mild cognitive impairment and normal cognition. *Neuroimage Clin.* 17 847–855. 10.1016/j.nicl.2017.12.013 29527489 PMC5842729

[B8] Elton A. Gao W. (2015). Task-related modulation of functional connectivity variability and its behavioral correlations. *Hum. Brain Mapp.* 36 3260–3272. 10.1002/hbm.22847 26015070 PMC6869497

[B9] Fiorenzato E. Strafella A. P. Kim J. Schifano R. Weis L. Antonini A. (2019). Dynamic functional connectivity changes associated with dementia in Parkinson’s disease. *Brain* 142 2860–2872. 10.1093/brain/awz192 31280293 PMC6736370

[B10] Franklin K. A. Lindberg E. (2015). Obstructive sleep apnea is a common disorder in the population-a review on the epidemiology of sleep apnea. *J. Thorac. Dis.* 7 1311–1322. 10.3978/j.issn.2072-1439.2015.06.11 26380759 PMC4561280

[B11] Fu Z. Tu Y. Di X. Du Y. Pearlson G. D. Turner J. A. (2018). Characterizing dynamic amplitude of low-frequency fluctuation and its relationship with dynamic functional connectivity: An application to schizophrenia. *Neuroimage* 180 619–631. 10.1016/j.neuroimage.2017.09.035 28939432 PMC5860934

[B12] Harper R. M. Macey P. M. Henderson L. A. Woo M. A. Macey K. E. Frysinger R. C. (1985). fMRI responses to cold pressor challenges in control and obstructive sleep apnea subjects. *J. Appl. Physiol.* 2003 1583–1595. 10.1152/japplphysiol.00881.2002 12514164

[B13] Hu G. Zhang Q. Waters A. B. Li H. Zhang C. Wu J. (2019). Tensor clustering on outer-product of coefficient and component matrices of independent component analysis for reliable functional magnetic resonance imaging data decomposition. *J. Neurosci. Methods* 325:108359. 10.1016/j.jneumeth.2019.108359 31306718

[B14] Huang X. Tang S. Lyu X. Yang C. Chen X. (2019). Structural and functional brain alterations in obstructive sleep apnea: A multimodal meta-analysis. *Sleep Med.* 54 195–204. 10.1016/j.sleep.2018.09.025 30580194

[B15] Jiang S. F. Shi J. Y. Yang Z. T. Zhang L. Chen H. J. (2020). Aberrant dynamic functional network connectivity in cirrhotic patients without overt hepatic encephalopathy. *Eur. J. Radiol.* 132:109324. 10.1016/j.ejrad.2020.109324 33038576

[B16] Johns M. W. (1991). A new method for measuring daytime sleepiness: The epworth sleepiness scale. *Sleep* 14 540–545. 10.1093/sleep/14.6.540 1798888

[B17] Kerns J. G. Cohen J. D. MacDonald A. R. Cho R. Y. Stenger V. A. Carter C. S. (2004). Anterior cingulate conflict monitoring and adjustments in control. *Science* 303 1023–1026. 10.1126/science.1089910 14963333

[B18] Kim J. Criaud M. Cho S. S. Díez-Cirarda M. Mihaescu A. Coakeley S. (2017). Abnormal intrinsic brain functional network dynamics in Parkinson’s disease. *Brain* 140 2955–2967. 10.1093/brain/awx233 29053835 PMC5841202

[B19] Kong L. Li H. Shu Y. Liu X. Li P. Li K. (2022). Aberrant resting-state functional brain connectivity of insular subregions in obstructive sleep apnea. *Front. Neurosci.* 15:765775. 10.3389/fnins.2021.765775 35126035 PMC8813041

[B20] Lee M. H. Yun C. H. Min A. Hwang Y. H. Lee S. K. Kim D. Y. (2019). Altered structural brain network resulting from white matter injury in obstructive sleep apnea. *Sleep* 42:z120. 10.1093/sleep/zsz120 31260533

[B21] Li C. Fronczek-Poncelet J. Lange D. Hennecke E. Kroll T. Matusch A. (2020). Impact of acute sleep deprivation on dynamic functional connectivity states. *Hum. Brain Mapp.* 41 994–1005. 10.1002/hbm.24855 31680379 PMC7268022

[B22] Li H. J. Dai X. J. Gong H. H. Nie X. Zhang W. Peng D. C. (2015). Aberrant spontaneous low-frequency brain activity in male patients with severe obstructive sleep apnea revealed by resting-state functional MRI. *Neuropsychiatr. Dis. Treat.* 11 207–214. 10.2147/NDT.S73730 25653530 PMC4311758

[B23] Li H. Li L. Shao Y. Gong H. Zhang W. Zeng X. (2016). Abnormal intrinsic functional hubs in severe male obstructive sleep apnea: Evidence from a voxel-wise degree centrality analysis. *PLoS One* 11:e164031. 10.1371/journal.pone.0164031 27723821 PMC5056709

[B24] Li H. J. Nie X. Gong H. H. Zhang W. Nie S. Peng D. C. (2016). Abnormal resting-state functional connectivity within the default mode network subregions in male patients with obstructive sleep apnea. *Neuropsychiatr. Dis. Treat.* 12 203–212. 10.2147/NDT.S97449 26855576 PMC4725694

[B25] Li H. Li L. Kong L. Li P. Zeng Y. Li K. (2021). Frequency specific regional homogeneity alterations and cognitive function in obstructive sleep apnea before and after short-term continuous positive airway pressure treatment. *Nat. Sci. Sleep* 13 2221–2238. 10.2147/NSS.S344842 34992481 PMC8714019

[B26] Li P. Shu Y. Liu X. Kong L. Li K. Xie W. (2022). The effects of CPAP treatment on resting-state network centrality in obstructive sleep apnea patients. *Front. Neurol.* 13:801121. 10.3389/fneur.2022.801121 35418931 PMC8995649

[B27] Liguori C. Maestri M. Spanetta M. Placidi F. Bonanni E. Mercuri N. B. (2021). Sleep-disordered breathing and the risk of Alzheimer’s disease. *Sleep Med. Rev.* 55:101375. 10.1016/j.smrv.2020.101375 33022476

[B28] Lin W. C. Hsu T. W. Lu C. H. Chen H. L. (2020). Alterations in sympathetic and parasympathetic brain networks in obstructive sleep apnea. *Sleep Med.* 73 135–142. 10.1016/j.sleep.2020.05.038 32827886

[B29] Liu F. Wang Y. Li M. Wang W. Li R. Zhang Z. (2017). Dynamic functional network connectivity in idiopathic generalized epilepsy with generalized tonic-clonic seizure. *Hum. Brain Mapp.* 38 957–973. 10.1002/hbm.23430 27726245 PMC6866949

[B30] Liu X. Chen L. Duan W. Li H. Kong L. Shu Y. (2022). Abnormal functional connectivity of hippocampal subdivisions in obstructive sleep apnea: A resting-state functional magnetic resonance imaging study. *Front. Neurosci.* 16:850940. 10.3389/fnins.2022.850940 35546892 PMC9082679

[B31] Lottman K. K. Kraguljac N. V. White D. M. Morgan C. J. Calhoun V. D. Butt A. (2017). Risperidone effects on brain dynamic connectivity-a prospective resting-state fMRI study in schizophrenia. *Front. Psychiatry* 8:14. 10.3389/fpsyt.2017.00014 28220083 PMC5292583

[B32] Luo Y. Qiao M. Liang Y. Chen C. Zeng L. Wang L. (2022). Functional brain connectivity in mild cognitive impairment with sleep disorders: A study based on resting-state functional magnetic resonance imaging. *Front. Aging Neurosci.* 14:812664. 10.3389/fnagi.2022.812664 35360208 PMC8960737

[B33] Malhi G. S. Das P. Outhred T. Bryant R. A. Calhoun V. (2019). Resting-state neural network disturbances that underpin the emergence of emotional symptoms in adolescent girls: Resting-state fMRI study. *Br. J. Psychiatry* 215 545–551. 10.1192/bjp.2019.10 30880661

[B34] Nasreddine Z. S. Phillips N. A. Bedirian V. Charbonneau S. Whitehead V. Collin I. (2005). The montreal cognitive assessment, MoCA: A brief screening tool for mild cognitive impairment. *J. Am. Geriatr. Soc.* 53 695–699. 10.1111/j.1532-5415.2005.53221.x 15817019

[B35] Olaithe M. Bucks R. S. Hillman D. R. Eastwood P. R. (2018). Cognitive deficits in obstructive sleep apnea: Insights from a meta-review and comparison with deficits observed in COPD, insomnia, and sleep deprivation. *Sleep Med. Rev.* 38 39–49. 10.1016/j.smrv.2017.03.005 28760549

[B36] Park B. Palomares J. A. Woo M. A. Kang D. W. Macey P. M. Yan-Go F. L. (2016). Disrupted functional brain network organization in patients with obstructive sleep apnea. *Brain Behav.* 6:e441. 10.1002/brb3.441 27099802 PMC4831421

[B37] Park H. R. Cha J. Joo E. Y. Kim H. (2022). Altered cerebrocerebellar functional connectivity in patients with obstructive sleep apnea and its association with cognitive function. *Sleep* 45:zsab209. 10.1093/sleep/zsab209 34432059 PMC8754484

[B38] Prilipko O. Huynh N. Schwartz S. Tantrakul V. Kim J. H. Peralta A. R. (2011). Task positive and default mode networks during a parametric working memory task in obstructive sleep apnea patients and healthy controls. *Sleep* 34 293–301. 10.1093/sleep/34.3.293 21358846 PMC3041705

[B39] Seeley W. W. Menon V. Schatzberg A. F. Keller J. Glover G. H. Kenna H. (2007). Dissociable intrinsic connectivity networks for salience processing and executive control. *J. Neurosci.* 27 2349–2356. 10.1523/JNEUROSCI.5587-06.2007 17329432 PMC2680293

[B40] Senaratna C. V. Perret J. L. Lodge C. J. Lowe A. J. Campbell B. E. Matheson M. C. (2017). Prevalence of obstructive sleep apnea in the general population: A systematic review. *Sleep Med. Rev.* 34 70–81. 10.1016/j.smrv.2016.07.002 27568340

[B41] Shang J. Lui S. Meng Y. Zhu H. Qiu C. Gong Q. (2014). Alterations in low-level perceptual networks related to clinical severity in PTSD after an earthquake: A resting-state fMRI study. *PLoS One* 9:e96834. 10.1371/journal.pone.0096834 24823717 PMC4019529

[B42] Soheili-Nezhad S. Jahanshad N. Guelfi S. Khosrowabadi R. Saykin A. J. Thompson P. M. (2020). Imaging genomics discovery of a new risk variant for Alzheimer’s disease in the postsynaptic SHARPIN gene. *Hum. Brain Mapp.* 41 3737–3748. 10.1002/hbm.25083 32558014 PMC7416020

[B43] Song X. Roy B. Vacas S. Woo M. A. Kang D. W. Aysola R. S. (2022). Brain regional homogeneity changes after short-term positive airway pressure treatment in patients with obstructive sleep apnea. *Sleep Med.* 91 12–20. 10.1016/j.sleep.2022.02.005 35245787 PMC10498724

[B44] Sridharan D. Levitin D. J. Menon V. (2008). A critical role for the right fronto-insular cortex in switching between central-executive and default-mode networks. *Proc. Natl. Acad. Sci. U.S.A.* 105 12569–12574. 10.1073/pnas.0800005105 18723676 PMC2527952

[B45] Vanek J. Prasko J. Genzor S. Ociskova M. Kantor K. Holubova M. (2020). Obstructive sleep apnea, depression and cognitive impairment. *Sleep Med.* 72 50–58. 10.1016/j.sleep.2020.03.017 32544796

[B46] Wang C. Cai H. Sun X. Si L. Zhang M. Xu Y. (2020). Large-scale internetwork functional connectivity mediates the relationship between serum triglyceride and working memory in young adulthood. *Neural Plast.* 2020:8894868. 10.1155/2020/8894868 33204252 PMC7652625

[B47] Wang P. Wang J. Michael A. Wang Z. Klugah-Brown B. Meng C. (2022). White matter functional connectivity in resting-state fMRI: Robustness, reliability, and relationships to gray matter. *Cereb. Cortex* 32 1547–1559. 10.1093/cercor/bhab181 34753176

[B48] Xue K. Liang S. Yang B. Zhu D. Xie Y. Qin W. (2020). Local dynamic spontaneous brain activity changes in first-episode, treatment-naive patients with major depressive disorder and their associated gene expression profiles. *Psychol. Med.* 30, 1–10. 10.1017/S0033291720003876 33121546

[B49] Yan L. Park H. R. Kezirian E. J. Yook S. Kim J. H. Joo E. Y. (2021). Altered regional cerebral blood flow in obstructive sleep apnea is associated with sleep fragmentation and oxygen desaturation. *J. Cereb. Blood Flow Metab.* 41 2712–2724. 10.1177/0271678X211012109 33906511 PMC8504950

[B50] Yao Z. Shi J. Zhang Z. Zheng W. Hu T. Li Y. (2019). Altered dynamic functional connectivity in weakly-connected state in major depressive disorder. *Clin. Neurophysiol.* 130 2096–2104. 10.1016/j.clinph.2019.08.009 31541987

[B51] Zhang Q. Qin W. He X. Li Q. Chen B. Zhang Y. (2015). Functional disconnection of the right anterior insula in obstructive sleep apnea. *Sleep Med.* 16 1062–1070. 10.1016/j.sleep.2015.04.018 26298780

[B52] Zhang Q. Wang D. Qin W. Li Q. Chen B. Zhang Y. (2013). Altered resting-state brain activity in obstructive sleep apnea. *Sleep* 36 651–659. 10.5665/sleep.2620 23633747 PMC3624819

[B53] Zhao C. Huang W. J. Feng F. Zhou B. Yao H. X. Guo Y. E. (2022). Abnormal characterization of dynamic functional connectivity in Alzheimer’s disease. *Neural Regen. Res.* 17 2014–2021. 10.4103/1673-5374.332161 35142691 PMC8848607

[B54] Zhou C. Gao T. Guo T. Wu J. Guan X. Zhou W. (2020). Structural covariance network disruption and functional compensation in Parkinson’s disease. *Front. Aging Neurosci.* 12:199. 10.3389/fnagi.2020.00199 32714179 PMC7351504

