# Supplementary material for: Abnormal dynamic functional network connectivity in male obstructive sleep apnea with mild cognitive impairment: A data-driven functional magnetic resonance imaging study
Source: Front Aging Neurosci. 2022 Oct 25;14:977917. doi: 10.3389/fnagi.2022.977917 (PMC9640755; doi:10.3389/fnagi.2022.977917)
Supplement: Supplementary file 1 [file Data_Sheet_1.doc]

**Supplementary materials**

| **Supplementary Table 1** MRI scanning parameters | | | | | | | | |
| --- | --- | --- | --- | --- | --- | --- | --- | --- |
| scanning sequence | TR, ms | TE, ms | thickness, mm | gap, mm | FOV, mm^2^ | matrix | slices | orientation |
| T2-weighted imaging | 4000 | 113 | 5 | 1.5 | 220 × 220 | 64 | 19 | axial |
| T1-weighted imaging | 250 | 2.46 | 5 | 1.5 | 220 × 220 | 64 | 19 | axial |
| Three-dimensional T1-weighted imaging | 1900 | 2.26 | 1 | 0.5 | 250 × 250 | 128 | 176 | sagittal |
| BOLD | 2000 | 30 | 4 | 1.2 | 230 × 230 | 64 | 30 | axial |
| Note: TR, repetition time; TE, echo time; BOLD, blood oxygen level-dependent. | | | | | | | | |

| **Supplementary Table 2** Peak coordinates of all ICs | | | | |
| --- | --- | --- | --- | --- |
| IC Regions | Tmax | Peak coordinate (mm) | | |
|  |  | X | Y | Z |
| **Auditory Network** |  |  |  |  |
| IC42 Bi Superior Temporal Gyrus | 28.8 | -48.5 | -18.5 | 9.5 |
| **Default Mode Network** |  |  |  |  |
| IC2 Bi Cuneus | 30.4 | -0.5 | -83.5 | 38.5 |
| IC12 Precuneus | 30.6 | -2.5 | -69.5 | 36.5 |
| IC14 Posterior Cingulate Cortex, Bi Inferior Parietal Lobule | 39.7 | 0.5 | -51.5 | 29.5 |
| IC18 Medial Prefrontal Cortex | 33.9 | -2.5 | 33.5 | -8.5 |
| IC36 Bi Precuneus | 31.4 | -12.5 | -65.5 | 17.5 |
| **Executive Control Network** |  |  |  |  |
| IC9 Bi Middle Frontal Gyrus | 25.5 | -57.5 | -8.5 | 33.5 |
| IC10 Bi Superior Parietal Lobule | 27 | -27.5 | -56.5 | 57.5 |
| IC22 Bi Lateral Occipital Cortex | 33.5 | 38.5 | -78.5 | 33.5 |
| IC28 L Lateral Occipital Cortex | 35.5 | -41.5 | -71.5 | 42.5 |
| IC33 L Middle Frontal Gyrus | 27.5 | -47.5 | 11.5 | 32.5 |
| IC41 R Lateral Occipital Cortex | 36.5 | 50.5 | -45.5 | 51.5 |
| **Language Network** |  |  |  |  |
| IC17 L Supramarginal Gyrus | 22 | -53.5 | -32.5 | 44.5 |
| IC32 R Supramarginal Gyrus | 30.8 | 59.5 | -48.5 | 23.5 |
| **Sensorimotor Network** |  |  |  |  |
| IC35 Bi Postcentral Gyrus | 27.5 | 42.5 | -32.5 | 53.5 |
| IC39 Bi Precentral Gyrus | 32.1 | 62.5 | -29.5 | 29.5 |
| **Salience Network** |  |  |  |  |
| IC19 Bi Insular | 31.8 | -35.5 | 11.5 | -24.5 |
| IC24 Dorsal Anterior Cingulate Cortex | 37.5 | -0.5 | 14.5 | 47.5 |
| IC44 Middle Cingulate Gyrus | 33.4 | -21.5 | -32.5 | 65.5 |
| **Visual Network** |  |  |  |  |
| IC6 Bi Lingual | 30 | 6.5 | -92.5 | -2.5 |
| IC13 Bi Lateral Occipital Cortex | 25.7 | 41.5 | -83.5 | 2.5 |
| IC16 Bi Cuneus | 33.8 | 17.5 | -93.5 | 11.5 |
| IC25 R Fusiform Gyrus | 25 | 23.5 | -88.5 | -10.5 |
| IC30 L Fusiform Gyrus | 24 | -23.5 | -86.5 | -12.5 |
| **Cerebellar Network** |  |  |  |  |
| IC23 Bi Cerbellar Posterior Lobe | 30.6 | 30.5 | -68.5 | -26.5 |
| L, left; R, right; Bi, bilateral. | | | | |

**Supplementary**
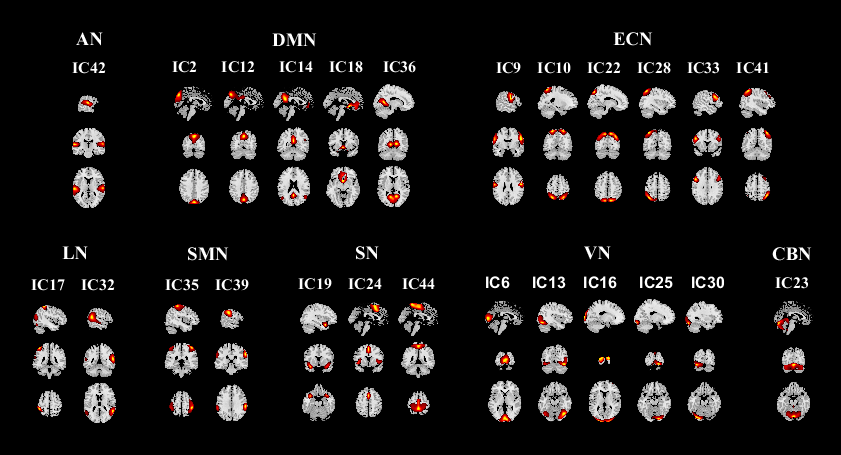
 **Figure 1** Spatial maps of functional independent components was divided into eight different functional domains, namely, AN, DMN, ECN, LN, SMN, SN, VN and CBN. AN, auditory network; DMN, default mode network; ECN, executive control network; LN, language network; SMN, sensorimotor network; SN, salience network; VN, visual network; CBN, cerebellum network.
